# Natural Experiments as a Study Method in Spinal Trauma Surgery: A Systematic Review

**DOI:** 10.1177/21925682231220889

**Published:** 2023-12-11

**Authors:** Agnita Stadhouder, Luke Xander van Rossenberg, Charlotte Kik, S. P. J. Muijs, F. C. Öner, R. Marijn Houwert

**Affiliations:** 1 Department of Orthopaedics and Sports Medicine, 26066 Amsterdam University Medical Centers, Amsterdam, Netherlands; 2 Faculty of Health Sciences and Medicine, 30731 University of Lucerne, Lucerne, Switzerland; 3 Department of Trauma Surgery, Diakonessenhuis, Utrecht, Netherlands; 4 Department of Neurosurgery, 6993 Erasmus MC, Rotterdam, Netherlands; 5 Department of Orthopaedics, 8124 University Medical Center Utrecht, Utrecht, Netherlands; 6 Department of Trauma Surgery, 8124 University Medical Center Utrecht, Utrecht, Netherlands

**Keywords:** trauma, methodology, natural experiment, systematic review, spinal fracture

## Abstract

**Study Design:**

Systematic review.

**Objectives:**

To determine if the natural experiment design is a useful research methodology concept in spinal trauma care, and to determine if this methodology can be a viable alternative when randomized controlled trials are either infeasible or unethical.

**Methods:**

A Medline, Embase and Cochrane database search was performed between 2004 and 2023 for studies comparing different treatment modalities of spinal trauma. All observational studies with a natural experiment design comparing different treatment modalities of spinal trauma were included. Data extraction and quality assessment with the MINORS criteria was performed.

**Results:**

Four studies with a natural experiment design regarding patients with traumatic spinal fractures were included. All studies were retrospective, one study collected follow-up data prospectively. Three studies compared different operative treatment modalities, whereas one study compared different antibiotic treatment strategies. Two studies compared preferred treatment modalities between expertise centers, one study between departments (neuro- and orthopedic surgery) and one amongst surgeons. For the included retrospective studies, MINORS scores (maximum score 18) were high ranging from 12-17 and with a mean (SD) of 14.6 (1.63).

**Conclusions:**

Since 2004 only four studies using a natural experiment design have been conducted in spinal trauma. In the included studies, comparability of patient groups was high emphasizing the potential of natural experiments in spinal trauma research. Natural experiments design should be considered more frequently in future research in spinal trauma as they may help to address difficult clinical problems when RCT’s are infeasible or unethical.

## Introduction

In current evidence based medicine practice, randomized controlled trials are considered the gold standard, as this methodology is particularly effective in preventing selection bias, information bias and confounding.^1–3^ However, randomized controlled trials in surgical fields may encounter certain difficulties which reduce reliability of results and allow introduction of bias.^4–6^ Practical difficulties such as a learning curve for new procedures, variation in quality of surgical performance or clinician and patient equipoise are common in general surgical studies.^7–11^ Methodical difficulties include challenges in acquiring informed consent, blinding of patients and randomization of patients.^9,11,12^ In acute surgery fields, where urgent lifesaving treatment is often involved, randomized controlled trials are difficult to conduct properly due these challenges. This is also the case for spinal trauma care.^10,13^ Other study designs might pose a more viable solution.^9^

Observational studies have historically been used to demonstrate credible results in situations where a randomized controlled trial is either unethical or unfeasible.^14^ However, observational studies are more prone to bias and confounding.^14–16^ To minimize confounding, observational studies must be carefully and rigorously designed.^17^ In therapeutic studies a randomized design has greater value and credibility of results compared to observational studies and Vandenbroucke^17^ states that observational studies will be credible only in exceptional circumstances. To ensure similar credibility of observational studies compared to randomized studies, three essential restrictions have been proposed by Vandenbroucke^17^ in the Lancet in 2004.^17^ The first restriction pertains to the selection of research topics where allocation of exposure is minimally associated with the outcome of interest. This is the most easily applicable in studies on adverse events as these are always unintended and their risk unknown or unpredictable. The second restriction involves that a study design is required to have at least a quasi-random allocation of exposure to treatment. Quasi-random allocation is a method of allocating participants which is not fully random or blinded but prevents researcher/clinician biased allocation of treatment based on patients characteristics or prognosis.^17,18^ Examples of quasi random allocation include allocation by date of birth or geographical location. The third is restriction to topics where potential confounding variables can be identified, accurately measured, and appropriately adjusted for in statistical models.

Among the different types of observational studies, the natural experiment is a promising method that mimics the design of an RCT without the need for randomization. As described by van de Wall et al “A natural experiment is a quasi-experimental study in which patients are exposed to either the experimental or control condition, whereby treatment allocation is determined by factors outside the control of the investigators.”^18^ To ensure adequate comparability, it is crucial that a genuine state of clinical equipoise is present, where both treatment strategies are considered equally viable options.^19^ Clinical equipoise is “a state of genuine uncertainty on the part of the clinical investigator regarding the comparative therapeutic merits of different treatment options”^20^ Clinical equipoise resulting in different treatment strategies can occur on various levels, e.g. amongst surgeons, hospitals, expertise centers, or so called “schools”, as well as internationally.^21^ A natural experiment becomes feasible when clinical equipoise is present and allocation of treatment is dependent on external factors.^21^ This is especially true for trauma patients. Generally, trauma patients will receive acute care at the nearest hospital able to facilitate adequate treatment.^22,23^ In this case allocation of treatment is determined by the geographical location of the incident, rather than by patient characteristics or any manipulation of the researcher. The hospital where the patient is treated determines the exposure to either the control or experimental condition and utilizing natural variation of treatment allocation increases validity of results as it emulates randomization.^21^ Multiple natural experiments in trauma surgery have been conducted reporting results matching the credibility of randomized controlled trials.^24–28^ However, natural experiment design is a relatively new study method in surgical research. This is also the case in spinal trauma and it is currently unknown to what degree natural experiment designs are utilized and to what extent they provide credible evidence.

Therefore, this systematic review aims to investigate to what extent natural experiment design has been conducted in all types of spinal trauma, and if they pose a viable alternative for randomized controlled trials in this field.

## Methods

### Search Strategy

This study was conducted in line with the PRISMA guidelines. We systematically searched literature on primary intervention studies reporting on natural experiments in spinal trauma patients. The systematic search was performed from 2004 until 2023 and updated on the 30^st^ of March using the search terms ‘spinal trauma’, ‘spinal fractures’, ‘vertebrae’ and synonyms in the Medline, Embase and the Central databases. Full text, English or Dutch written articles were reviewed for inclusion. The full search string is provided in Appendix A.

### Study Selection and Eligibility Criteria

Three reviewers (AS, SC, LXR) independently assessed the titles and abstracts to identify cohort studies with a natural experiment design in adult spinal trauma patients. A study was considered a natural experiment if there was evident geographical (pseudo)randomization of treatment allocation, either amongst schools, departments or surgeons. (e.g., surgeon A always performs a certain type of treatment, whilst surgeon B always performs a different type of treatment for similar injuries). Historic comparison studies were excluded since in a certain time span of the research period also other factors can be changed.

Subsequently, full texts were independently evaluated for eligibility following in- and exclusion criteria, which are displayed in Table 1. Disagreement was resolved through consensus. Non-English or Dutch reports, randomized controlled trials, systematic reviews, and meta-analyses, reviews, cohorts with a historical control, case-control studies, cross-sectional studies, case reports, case series, conference abstracts, editorials, letters and comments and animal studies were excluded. EndNote X8 was used to manage the screening and reviewing process. Finally, the reference lists of included articles and relevant reviews were screened for eligible studies.

**Table 1.** Summary of Inclusion and Exclusion Criteria.

Inclusion Criteria		Exclusion Criteria	
Population	All patients with a traumatic spinal fracture (total spine)	Population	Malignant or osteoporotic fractures
Intervention/comparison	Comparison between any two treatments in spinal trauma surgery	Intervention/comparison	No spinal treatment
Outcome	Not applicable	Outcome	Not applicable
Study design	Observational study with natural experiment as study design	Study design	Historic comparison
			Other study design^a^

NE, natural experiment.

^a^(RCT, case series, case control, case report, observational cohort studies without NE* design).

### Data Extraction

Two investigators (AS and LXR) extracted data independently of all included studies. From each eligible study, the following data were collected: first author, year of publication, country of conduct, study design, number of included spinal trauma patients, number of patients in the intervention group, number of patients in the control group, mean age of participants, gender and the mean Injury Severity Score (ISS) if available.

### Quality Assessment

The methodologies of the included studies were critically appraised using the validated Methodological Index for Non-Randomized Studies (MINORS) criteria, which assesses articles on the presence of various forms of bias including selection, performance, detection, attrition, reporting and other bias (scored as ‘not reported’, ‘reported but inadequate’ or ‘reported adequately’).^29^ Two authors (AS and LXR) scored all articles independently. When in disagreement, a third reviewer (CK) was asked to make an additional assessment and the majority vote was counted. A maximum of 24 points could be given to prospective comparative studies, and 18 points for retrospective comparative studies, as MINORS criteria “prospective collection of data”, “loss to follow up less than 5%” and “prospective calculation of the study size” are not applicable for retrospective designs. Retrospective comparative studies with MINORS scores ranging between 12 and 18 are considered high quality.^30^ Further information on the assessment of methodological quality is provided in Appendix B.

## Results

### Identification of Studies

**Figure 1.**
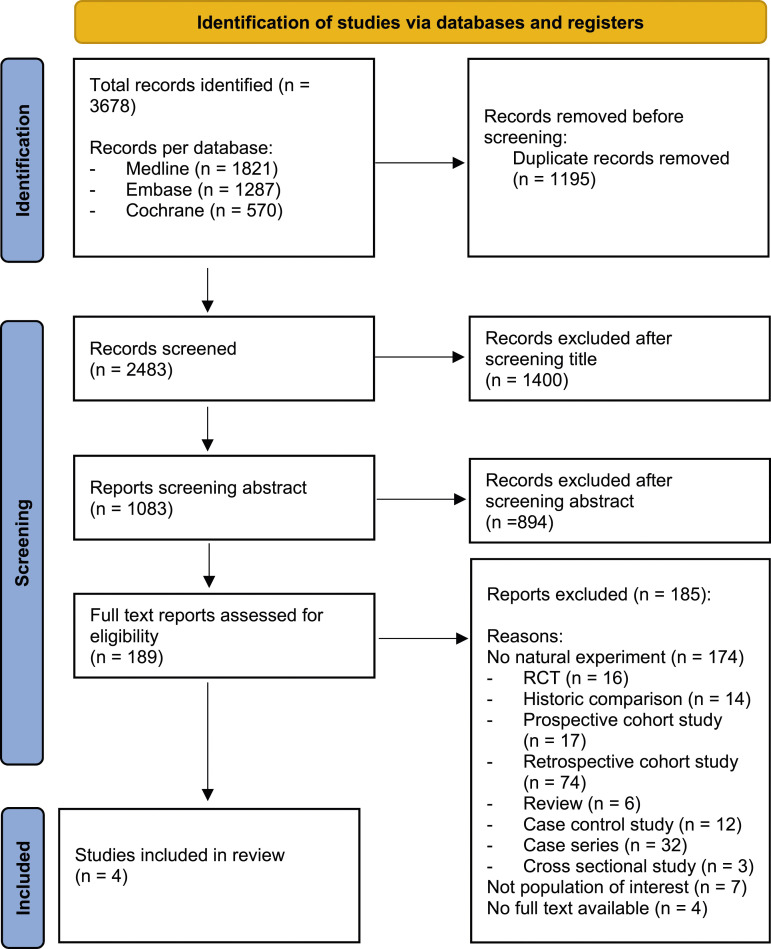
Flowchart of study selection. *From:* Page MJ, McKenzie JE, Bossuyt PM, Boutron I, Hoffmann TC, Mulrow CD, et al The PRISMA 2020 statement: an updated guideline for reporting systematic reviews. BMJ 2021;372:n71. doi: 10.1136/bmj.n71.

The systematic search yielded 3678 articles. After removal of duplicates, 2483 articles were screened on title and abstract for eligibility. One-hundred and eighty-nine citations were retrieved for full-text assessment and evaluated for inclusion. One-hundred and eighty-five studies were excluded because they did not adhere to the desired “natural-experiment” design, outlined in Figure 1.

### Study Characteristics

The overall characteristics of the included studies are summarized in Table 2. Studies were published between 2008 and 2021.^13,31–33^ All four studies were designed retrospectively,^13,31–33^ of which one study retrieved participant retrospectively but collected patient reported outcomes actively of included participants.^13^ Three studies were performed in Western Europe,^13,31,32^ while one was conducted in the United States of America.^33^ Two studies included a multi-center setting.^13,31^ All participating hospitals in the studies were level-1 trauma centers.^13,31–33^ Three studies included a comparison between different surgical treatment modalities for acute spinal fractures,^13,31,32^ while one study focused on the rate of infectious complications by the use of vancomycin powder in posterior spinal stabilization of traumatic injuries.^33^ Of the 852 participants in the included studies 481 (56%) were male, and the mean age ranged from 37 to 69 years.^13,31–33^ Mean follow-up ranged from 6 months to 74 months.^13,31–33^ Two studies reported the injury severity score (ISS) of which all participants had mean ISS scores ≤ 16.^31,32^ Three studies reported trauma mechanisms and of the 742 participants the majority was injured due to a fall (78.8%), the minority by traffic accident (11.5%) or other specific causes (9.2%) (e.g., paragliding, horse riding or skiing).^13,31,32^ Of all spinal trauma injuries, the majority of patients had either a thoracic and/or lumbar fracture (66.2%), whereas cervical fractures were less common (33.8%).^13,31–33^ Three studies reported presence of neurological impairment, of all 765 patients 157 (20.5%) were partially or completely neurologically impaired.^13,31,32^ One study excluded patients with cervical fractures and/or neurological impairment.^33^

**Table 2.** Baseline Characteristics of Included Studies With a Natural Experiment Design.

Study and Year	Study Period	Design	Country	Natural Experiment Comparison^a^	Number of Patients		Age in Years, Mean (SD/Range)	Gender Male, n (%)	Follow Up in Months, Mean (SD/Range)	Mean (SD) ISS^b^	Trauma Mechanism, n (%)			Fracture Location, n (%)		Neurological Impairment, n (%)
											Fall	Traffic Accident	Other	Cervical	Thoracolumbar	
					Total	Case/Control	Case/Control	Case/Control	Case/Control	Case/Control	Case/Control	Case/Control	Case/Control	Case/Control	Case/Control	Case/Control
Myers, 2021	2012-2016	Retrospective	United Kingdom	Departments, SS	465	266/199	60.2 (21.3)/61/1 (22)	146 (54.9%)/106 (53.3%)	*na*	8.7 (4.6)/8.9 (6.9)	446 (95.9%)^c^	15 (3.2%)^c^	4 (0.8%)^c^	128 (20.8%)/106 (17.2%)	229 (37.2%)/152 (24.7%)	27 (10.2%)/20 (10%)
Erichsen, 2020	2013-2015	Retrospective	Switzerland/Germany	Schools, MS	87	44/43	43.5 (14.3)/48.4 (12.2)	19 (43.2%)/26 (60.5%)	24 (no SD)^c^	ISS ≤ 16 (no SD)^c^	21 (47.7%)/30 (69.8%)	9 (20.4%)/11 (25.5%)	14 (31.8%)/2 (4.6%)	*Excluded*	87 (100%)^c^	*Excluded*
O’Neill, 2011	2008-2009	Retrospective	United States	Surgeons, SS	110	56/54	43 (17)/45 (18)	35 (63%)/35 (65%)	6 (no SD)/7 (no SD)	*na*	*na*	*na*	*na*	21 (38%)/23 (43%)	35 (62%)/31 (57%)	22 (40%)/28 (52%)
Stadhouder, 2008	1991-2002	Retrospective	The Netherlands	Schools, MS	190	95/95	38.5 (18-84)/37.1 (18-79)	50 (52%)/64 (67%)	74.4 (no SD)^c^	*na*	46 (48%)/42 (44%)	27 (28%)/26 (27%)	22 (24%)/27 (29%)	61 (32.1%)^c^	63 (33.2%)/66 (34.7%)	23 (25%)/37 (39%)

Na, not reported/not measured; Excluded, patients with this characteristic were excluded for the study.

^a^What level of comparison (high to low = schools/expertise centers, departments, surgeons), SS single center, MS, multicenter.

^b^Injury severity score.

^c^Only overall value available, no distinction between case and control groups.

### Quality Assessment

On average total MINORS score for the four retrospective studies were high ranging from 12 to 17 with a mean (SD) of 14.6 (1.63). Stadhouder et al scored highest with a score of 17,^13^ followed by O’Neill et al and Erichsen et al both with score of 16.^31–33^ Myers et al scored lowest with a score of 12.^32^ Average scores in the MINORS section “additional scores for comparative studies” (range 0-8) were high, ranging from 6 to 8 with a mean (SD) of 7.25 (.75).^13,31–33^ See Table 3 for an overview of individual scores.

**Table 3.** MINORS Score.

MINORS Quality Assessment of Included Studies in a Systematic Review of Natural Experiments in Spinal Trauma Surgery				
Criteria	Stadhouder et al 2008	O’Neill et al 2011	Erichsen et al 2020	Myers et al 2021
A clearly stated aim	2	2	2	1
Inclusion of consecutive patients	1	2	2	2
Prospective collection of data^a^	0	0	0	0
Endpoints appropriate to the aim of the study	2	2	2	2
Unbiased assessment of the study endpoint	2	0	0	0
Follow-up period appropriate to the aim of the study	2	2	2	0
Loss to follow-up less than 5%^a^	0	0	0	0
Prospective calculation of the study size^a^	0	0	0	0
Additional criteria for comparative studies				
An adequate control group	2	2	2	2
Contemporary groups	2	2	2	2
Baseline equivalence of groups	2	2	1	2
Adequate statistical analyses	2	2	1	1
Total MINORS score	17	16	16	12

All items are scored 0 (not reported/not applicable), 1 (reported but inadequate) or 2 (reported and adequate).

^a^All included studies are retrospectively designed, scores range from 0-24 for comparative studies and 0-18 for retrospective comparative studies.

### The Natural Experiment Design

O’Neill et al used a natural experiment design to evaluate the effectiveness of perioperative intrawound vancomycin powder use in patients who underwent posterior spine fusion to prevent infections. Retrospective identification of patients over a 2-year period at a single academic center resulted in two groups: those who received vancomycin powder in their surgical wound during their initial surgery and those who did not, following the standard of care at the time. Patients were (pseudo)randomized by surgeon preference and only one surgeon always treated patient with intrawound vancomycin, whereas other surgeons did not. The study found that the use of intrawound vancomycin significantly reduced the incidence of infections in patients with traumatic spine injuries. Data retrieval began in 2004, and the study was published in 2011.^33^ The study demonstrated the possibility to use a natural experiment design in pharmaceutical studies.

Similarly, Stadhouder et al conducted a study on the operative vs nonoperative treatment of traumatic thoracic and lumbar spinal fractures using a natural experiment design. A blinded panel of orthopedic surgeons from two University Medical Centers retrospectively reviewed cases where there was disagreement on the suggested treatment modality, creating two comparable groups of patients who either underwent nonoperative or operative treatment. After an average follow-up period of 6 years, patients’ clinical outcomes were compared, and it was found that operative and nonoperative treatments were comparable. Start of data retrieval was 2004, publication year 2008. A limitation of the study was its retrospective design and longer follow up between treatment and outcome, which led to probable missing data. The authors suggested that a natural experiment design could also be used in prospective series.^13^ The study demonstrated the possibility of using clinical equipoise to create two comparable groups and compare treatment outcomes without influencing the treatment preferences of the surgeon/school.

In subsequent years, the natural experiment design has been utilized in two further studies related to the management of spinal fractures.^31,32^ Erichsen and colleagues conducted a retrospective review of cases involving patients with a traumatic AO spine type A3 fracture of the thoracolumbar spine who received different types treatment depending on which hospital they were treated. In one hospital all patients received open posterior stabilization, while in the other hospital all patients underwent percutaneous posterior stabilization. The treatment effects were evaluated after a 2-year follow-up period using the Owestry Disability Index, Visual Analog Scale, and a 36-item Short Form Health Survey. The trial was registered in the German Clinical Trial Registry in 2018, publication was in 2020.^31^ Similarly, Myers and colleagues conducted a retrospective evaluation of the difference in direct treatment outcomes between patients with spinal fractures who were treated by neurosurgical teams vs those treated by orthopedic teams in weekly shifts. The end of data retrieval period was December 2016, publication was in 2021.^32^ Both research groups had similar baseline characteristics, admittance practice strategies and exclusion rates.^31,32^ The authors conclude that the study demonstrates that the natural experiment design is suitable for comparing patient outcome between two different surgical specialties (schools) in the same hospital.^31,32^

## Discussion

In this systematic review on the methodology of natural experiments in spinal trauma, only four papers were found that used this methodology in 18 years of spinal trauma research.^13,31–33^ Topics of the four included papers differed: open vs percutaneous placement of pedicle screws in A3 fractures, differences in management of isolated spinal fractures between neurosurgeons and orthopedic surgeons on call, the use of intrawound vancomycin powder to reduce surgical site infections in spinal trauma posterior fixation and operative vs non-operative treatment in thoracolumbar spinal fractures.^13,31–33^ These are all relevant topics but in the spinal trauma community one can think of several other issues where clinical equipoise exists. Examples include conservative or operative treatment of AO classification A3 or A4 fractures,^34^ treatment strategy of C2 fractures in the elderly^35^ and timing of intervention in patients with spinal cord injury.^36^ For this matter natural experiments can be of value since within spinal trauma treatment, the different schools and treatments are common practice already.^37–40^ Therefore, with this paper we aim to increase the knowledge within the spinal community about natural experiments design and its promising potential in clinically meaningful research.

The development of prospective trauma databases can be an added value in performing natural experiments.^41^ As are the common practice of Electronic Medical Records (EMR) in hospitals,^42,43^ and Patient Reported Outcome Measurements (PROMS) prospectively gathered in specific patient groups.^44^ In the included retrospective natural experiment study of Stadhouder et al^13^ demographic and clinical data were not up to date. This was mostly due to the longer follow up period of 2-12 years. Gathering clinical and follow up data in a retrospective manner required a huge effort leading to a follow-up percentage of 79%. The longer follow up period can lead to attrition bias when the number of drop outs differ between the two groups. With longer follow up the number of dropouts will increase but there is no recognized dropout rate that is considered acceptable.^45^ For analysis of results of natural experiments, as in RCT data, there is no accepted specific strategy that deals with drop outs or loss to follow up.^45^ Results therefore should be carefully interpreted when there is a high and difference between groups number of drop outs.^45,46^

A study performed by the Canadian Orthopedic Trauma Society showed that the average time of presentation of concept to presentation of an RCT took almost 10 years.^47^ A review by Leatherdale et al on natural experiments in the public health domain, where natural experiments are more common, mentioned that one of the three core strengths of natural experiments is ‘creating timely evidence’.^48^ Van de Wall also noted that one of the differences between randomized clinical trials and natural experiments/traditional observational studies is that the latter are often fast in their time frame since most patients are already included.^18^ We observed in the included papers that the average time from data gathering to publication in the four studies included was 3.75 years (3-5 years).^13,31–33^ A shorter duration of study time can be a contributing factor to conducting research in quickly developing specialties as orthopedic trauma and spine surgery.^18,49^

Two included papers were published more than 10 years ago,^13,33^ two papers more recently.^31,32^ We think that natural experiments in clinical situations where equipoise is present have a promising future in trauma research. In this sense the total amount of four papers published utilizing some form of natural experiment in spinal trauma is disappointing. A possible explanation can be that this concept is not well known yet among spinal trauma researchers/surgeons. Another explanation might be that authors describe the method of a natural experiment inadequately, contributing to the difficulty of identifying a natural experiment. The Natural Experiments Study Group (NEXT Study Group) is an international non-profit collaboration of clinical researchers in the field of emergency and (orthopedic) trauma surgery.^50^ They so far published four relevant papers with a natural experiment as methodology and more studies are being conducted and soon to be published.^27,28,51,52^ One study showed that with a natural experiment design on rib fixation there was no difference in outcome between nonoperative and operative treated patients.^27^ The inclusion was finished one year earlier than predicted and took three years.^27^ Before this publication, a RCT was conducted in Australia which took four years and where almost half of the eligible patients refused to participate in this study.^53^ It shows the difficulties of conducting RCT’s in a trauma/surgery patient population. Also, the result showed no difference in outcome between operative and nonoperative patients,^53^ comparable with the natural experiment paper.^27^ Both articles impacted the current clinical practice in our hospital and resulted in an 80% decrease of surgical rib fixations. Currently surgical rib fixation is only performed in case of traumatic flail chest injuries and/or when difficulties in the weaning process of mechanical ventilation are present.

The MINORS criteria were developed as a methodological index for non-randomized studies to assess the quality of studies.^29^ It comprises twelve items with a maximum score of 24, that applies to meticulously designed RCT’s.^29^ The studies included in our paper had a score of respectively 12, 16, 16 and 17 points.^13,31–33^ Since all studies were retrospective, 3 of 4 studies were not blinded for outcome, and loss to follow up <5% is difficult to achieve in a trauma population, we consider the quality of the natural experiment studies high as compared to other non-natural experiment retrospective comparative studies.

A systematic review of 12 comparative studies published by Phan et al^54^ in 2015 on percutaneous vs open procedures in spinal fractures concluded that percutaneous screws were associated with shorter operative time and hospital stay, reduced intraoperative blood loss and reduced infection rates. They also stated that: “given the lack of robust clinical evidence, these findings warrant verification in large prospective registries and randomized trials.”^54^ Another more recent systematic review by Sathish et al evaluated 96 systematic reviews published in spine surgery.^55^ Reviews were scored by the AMSTAR score (A measurement Tool to Assess systematic Reviews),^56^ PRISMA (Preferred Reporting Items for Systematic Reviews and Meta-Analyses)^57^ and MOOSE (Meta-analyses Of Observational Studies in Epidemiology).^58^ The authors concluded that there is improvement in methodological quality of reviews and meta-analysis but a substantial proportion of critical flaws remain. To our opinion, this shows the difficulties in interpreting results of comparative studies and reviews in trauma and spinal surgery and one can argue if results of these studies have additive scientific value.

Natural experiments are more susceptible to confounding and bias, but when designed appropriately, it is possible to have robust internal and external validity and evidence.^48^ As stated in a previous published paper on natural experiments we suggest to collect data on key prognostic patient factors, either prospective or retrospective.^18^ Further, it is important to correct for confounding by stratification, regression adjustment or matching.^18^ Another solution is to use clinical equipoise as an inclusion criterion.^10,18–20,45,51^ Eligible data is presented to an independent expert panel, blinded for the actual treatment and the expert panel should be representative of the two schools that are compared.^10,18,51^ In this review one paper used an expert panel,^13^ the other three studies did not.^31–33^

When reviewing the 189 full text papers for inclusion in our review we noticed a high number of papers with a historical comparison group (see Figure 1). One of the MINORS criterion (No 10: Contemporary groups: control and study group should be managed during the same time period) considers a historical control group as less valid. This was also reported in a publication by Agabegi et al.^45^ They describe that historical controls should be used with caution because of differences in in- and exclusion criteria. Treatment techniques may have improved over time and results might be a reflection of this improvement instead of a treatment effect.^45^ Also it is unknown if patient and treatment factors study of controls and research subjects were similar in the time span of the study. We therefore excluded these studies.

To conclude, of the 2483 papers published on spinal trauma in the last 14 years only four papers had a natural experiment design. These papers were of high quality according to the MINORS criteria. This methodology has, to our opinion, a high potential in trauma and spinal trauma research to address difficult clinical problems in a relative short time span. We hope this systematic review will improve the attention for natural experiment designs in spinal trauma and trauma surgery.

## Supplemental Material

Supplemental Material - Natural Experiments as a Study Method in Spinal Trauma Surgery: A Systematic Review Supplemental Material for Natural Experiments as a Study Method in Spinal Trauma Surgery: A Systematic Review by Agnita Stadhouder, Luke Xander van Rossenberg, Charlotte Kik, S. P. J. Muis, F. C. Öner, and R. Marijn Houwert in Global Spine Journal.
