# Supplementary material for: Natural Experiments as a Study Method in Spinal Trauma Surgery: A Systematic Review
Source: Global Spine J. 2023 Dec 11;14(5):1640–9. doi: 10.1177/21925682231220889 (PMC11394511; doi:10.1177/21925682231220889)
Supplement: Supplemental Material - Natural Experiments as a Study Method in Spinal Trauma Surgery: A Systematic Review [file sj-pdf-1-gsj-10.1177_21925682231220889.pdf]

Appendix A – Full search string

**Supplementary materials table A.** Original search string (01-07-2021) and final update (30-03-2022)			
**Database**	**#**	**Syntax**	**Results**
**MEDLINE (n = 1821)**	1	((spinal or spine) adj (fracture* or injur*) adj3 (trauma* or burst)).ti,ab,kw.	827
	2	*Spinal Fractures/pp, su, th and (burst or trauma*).ti,ab,kw.	1832
	3	*Thoracic Vertebrae/in	2482
	4	1 or 2 or 3	4493
	5	exp Osteoporosis/ or osteoporos*.ti,ab,kw.	91707
	6	4 not 5	4135
	7	(exp Pediatrics/ or adolescent/ or exp child/ or exp infant/ or (child* or pediatr* or paediatr* or adolescen* or youth*).ti,ab,kw.) not exp Adult/	2355226
	8	6 not 7	3856
	9	exp Neoplasm Metastasis/ or metasta*.ti,ab,kw.	621026
	10	8 not 9	3818
	11	comment/ or editorial/ or letter/ or case reports/ or (letter or comment* or editorial or case report).ti.	4071872
	12	10 not 11	2855
	13	((exp Animals/ or exp Animal Experimentation/ or exp models, animal/ or (animal* or rat or rats or mice or mouse or dog or dogs or pig or pigs or swine or swines or cow or cows or monkey or monkeys or goat or goats or horse or horses).ti,ab,kw.) not (Humans/ or human*.ti,ab,kw.)) or animal*.ti.	4897417
	14	12 not 13	2780
	15	limit 14 to yr = "2004 -Current	1774
	16	limit 15 to (dutch or english or german)	1572
**Last update 30-3-2023**	1	orginal search repeated	1821
**EMBASE (n= 1287)**	1	((spinal or spine) adj (fracture* or injur*) adj3 (trauma* or burst)).ti,ab,kw.	1120
	2	*spine fracture/su, th and (burst or trauma*).ti,ab,kw.	1519
	3	1 or 2	2545
	4	exp osteoporosis/ or osteoporos*.ti,ab,kw.	170682
	5	3 not 4	2390
	6	exp metastasis/ or metasta*.ti,ab,kw.	1000273
	7	exp metastasis/ or metasta*.ti,ab,kw.	2374
	8	(exp child/ or juvenile/ or exp adolescent/ or exp infant/ or exp pediatrics/ or (child* or pediatr* or paediatr* or adolescen* or youth*).ti,ab,kw.) not (adult/ or exp aged/ or middle aged/)	3108037
	9	7 not 8	2207
	10	letter/ or editorial/ or note/ or case report/ or conference paper/ or (letter or comment* or editorial or case report).ti.	5885096
	11	9 not 10	1786
	12	(exp animal/ or exp animal experiment/ or exp animal model/ or (rat or rats or mice or mouse or dog or dogs or pig or pigs or cow or cows or monkey or monkeys or goat or goats or horse or horses or ape or apes or gorilla or gorillas).ti,ab,kw.) not (human/ or human*.ti,ab,kw.)	5738063
	13	11 not 12	1733
	14	limit 13 to yr="2004 -Current"	1359

	15	limit 14 to (dutch or english or german)	1202
	16	limit 14 to (dutch or english or german)	175
	17	15 not 16	1027
**Last update 30-3-2023**	1	orginal search repeated	1287
**CENTRAL (n= 570)**	1	((spinal or spine) near/3 (fracture* or injur*) near/3 (trauma* or burst)):ti,ab,kw	357
	2	MeSH descriptor: [Spinal Fractures] explode all trees and with qualifier(s): [physiopathology - PP, surgery - SU, therapy - TH]	295
	3	#1 or #2	640
	4	(osteoporos*):ti,ab,kw	11031
	5	#3 not #4	508
	6	(metasta*):ti,ab,kw	44239
	7	#5 not #6	504
	8	((child* or pediatr* or paediatr* or adolescen* or youth*) not adult*):ti,ab,kw	136919
	9	#7 not #8 in Trials	490
**Last update 30-3-2023**	1	orginal search repeated	570

**Supplementary table B.** Quality assessment in compliance with the MINORS criteria in a systematic review of natural experiments		
**Criteria**	**Reported and adequate (2)**	**Reported but in adequate (1)**
A clearly stated aim	Aim stated clearly	Aim unclear but reported
Inclusion of consecutive patients	Inclusion criteria and consecutive inlcusion reported	Inclusion criteria/consecutive inclusi unclear
Prospective collection of data*	Data collected prospectively and clearly reported	Prospective design, unclearly reporte
Endpoints appropriate to the aim of the study	Endpoints appropriate and reported clearly	Endpoints inappropriate or reported unclearly
Unbiased assessment of the study endpoint	Blinded assessment of outcomes	Reason for not blinding reported
Follow-up period appropriate to the aim of the study	Follow-up appropriate and reported clearly	Follow-up inappropriate or reported unclearly
Loss to follow-up less than 5%*	≤ 5% and reported	≥ 5% and reported
Prospective calculation of the study size*	Sample size calculation and power analysis performed	Only sample size or power analysis performed
**Additional criteria for comparative studies**		
An adequate control group	Natural experiment design	Not applicable (exclusion)
Contemporary groups	Groups managed in the same time period	Not applicable (exclusion)
Baseline equivalence of groups	Comparable baseline characteristics	Incomparable baseline characteristics

Appendix B – Minors screening criteria

Adequate statistical analyses	Statistical analysis reported and repeatable	Inadequately reported statistical analy
All items are scored 0 (not reported/not applicable), 1 (reported but inadequate) or 2 (reported and adequate). Overall scores of the M studies<br>*Overall scores for retrospective studies range from 0-18 as prospective collection of data, loss to follow-up and prospective calcula		
